# Body mass explains characteristic scales of habitat selection in terrestrial mammals

**DOI:** 10.1002/ece3.45

**Published:** 2011-12

**Authors:** Jason T Fisher, Brad Anholt, John P Volpe

**Affiliations:** 1 Alberta Innovates – Technology Futures, Ecosystem Management UnitVictoria, British Columbia, Canada; 2 University of Victoria, Department of BiologyVictoria, British Columbia, Canada; 3 Bamfield Marine Sciences CentreBamfield, British Columbia, Canada; 4 University of Victoria, School of Environmental StudiesVictoria, British Columbia, Canada

**Keywords:** Allometry, Habitat complexity, Spatial scale, Textural-discontinuity

## Abstract

Niche theory in its various forms is based on those environmental factors that permit species persistence, but less work has focused on defining the extent, or size, of a species’ environment: the area that explains a species’ presence at a point in space. We proposed that this habitat extent is identifiable from a characteristic scale of habitat selection, the spatial scale at which habitat best explains species’ occurrence. We hypothesized that this scale is predicted by body size. We tested this hypothesis on 12 sympatric terrestrial mammal species in the Canadian Rocky Mountains. For each species, habitat models varied across the 20 spatial scales tested. For six species, we found a characteristic scale; this scale was explained by species’ body mass in a quadratic relationship. Habitat measured at large scales best-predicted habitat selection in both large and small species, and small scales predict habitat extent in medium-sized species. The relationship between body size and habitat selection scale implies evolutionary adaptation to landscape heterogeneity as the driver of scale-dependent habitat selection.

## Introduction

Niche theory in its various forms (Chase and Leibold 2003) is based on those factors that permit species persistence. However, much less work has focused on defining the extent, or size, of a species’ environment: the area, or spatial scale, that predicts a species’ presence at a point in space. This scale describes the area that integrates an individual's lifetime interactions with competitors, predators, parasites, and mates (e.g., Wiens et al. 1993), and so serves as a spatial basis for studies of these ecological mechanisms.

Attempts to quantify this scale inevitably leads to the “scale problem” in ecology, wherein the selection of a scale of experimentation can radically affect the outcome of an experiment, a trait that tends to prevent reliable inference across systems (Levin 1992). Fundamental to scale theory are the two dimensions of scale: grain and extent. In ecological processes, grain is the limit of an organism's perception of heterogeneity, and extent is the maximum area affecting the organism. In analysis, grain refers to the lower limit of resolution, or “pixel size”; it is the smallest distinct unit of space (or time) being measured. Extent defines the size of the experimental unit. Kotliar and Wiens (1990) and Levin (1992) maintain that matching the grain and extent of an experiment to the grain and extent of the process it measures must be the first step in ecological inquiry. With the development of conceptual frameworks to solve the scale problem, such as scale domains (Wiens 1989) and ecological hierarchy theory (Allen and Hoekstra 1992), the concept of scale in ecology has developed from a pervasive cautionary tale (Levin 1992) to an increasingly effective scientific tool (Schneider 2001). Examination of ecological processes across scales has revealed much about these processes, from allometric relationships (Peters 1983) to biodiversity patterns (Storch et al. 2007).

For example, multiple-scale analysis of density-dependent habitat selection processes (Morris 1987; Morris 2003) and species–habitat relationship patterns (e.g., Fisher et al. 2005; Knegt et al. 2010b) show that habitat selection varies markedly across spatial scales. How and why it varies is hotly debated, due partly to inconsistencies in experimental design, data collection, and analysis among studies and species (Schooley 2006; Wheatley and Johnson 2009). It is therefore not surprising that recent attempts to derive generalities have highlighted the many controversies inherent in multiscale habitat selection (Bowyer and Kie 2006; Mayor et al. 2009a; Wheatley and Johnson 2009; Laca et al. 2010). It is unknown whether scale-dependent habitat selection is driven primarily by population-level responses (Morris 2003), individual responses that are a function of home-range size (e.g., McLoughlin et al. 2004), or both.

At the heart of these and other major conceptual gaps lays an unanswered question: are several spatial scales required to explain a species occurrence at a point in space, or is there a single best scale? Several lines of evidence suggest that ecological processes operate simultaneously at different domains of scale; this basic prediction from hierarchy theory is the basis for hierarchical habitat selection (Johnson 1980), wherein different processes, such as foraging and home-range selection, occur at different scales. Notably, as Levin (1992) suggested, not all scales are equal. It is possible that within a species, one scale may better describe habitat selection than other scales. Holland et al. (2004) termed this the *characteristic selection scale*. A characteristic selection scale has been demonstrated for fly parasitoids in boreal forests (Roland and Taylor 1997), forest beetles in mixedwood forests (Holland et al. 2004), grizzly bears (*Ursus arctos horribilis*; Nams et al. 2006), and marten (*Martes americana*; Mowat 2006). Though the existence of a characteristic scale of selection within species has been suggested by several empirical studies, it remains contentious (Holland et al. 2004; Bowyer and Kie 2006; Schooley 2006).

If characteristic spatial scales of habitat selection exist, these may arise from a species’ interaction with landscape patchiness and resource dispersion. The spatial structure of the landscape has been shown to affect the scale of habitat selection for caribou (Mayor et al. 2007, 2009b, Schaefer and Mayor 2007) and elephants (Knegt et al. 2010a, 2010b). Species interact with landscape spatial structure through combined processes of foraging, dispersal, and migration. Since these processes scale with body mass (Peters 1983), we hypothesize that the characteristic scale of a species’ habitat selection increases linearly as a function of its body mass. This hypothesis can be tested by examining habitat selection across scales of different-sized species, occurring in the same landscape, at the same time. We test this hypothesis by simultaneously surveying 12 mammal species sympatrically distributed in a mountain landscape, and modeling the habitat selection of each species across 20 spatial scales. We test whether a characteristic habitat selection scale exists for these mammal species, and whether this scale is predicted by species’ body mass.

## Methods

### Study area

Terrestrial mammals were sampled in the Foothills and Rocky Mountains of west-central Alberta, Canada, over a 6,400-km^2^ area. The entire Rocky Mountain portion of our study site lies within the Willmore Wilderness Park, a 4,600-km^2^ conservation area protected from forest harvesting, mining, seismic exploration, and roads. Topography is rugged; alpine areas are characterized by mountain meadows of herbs and shrubs. Subalpine slopes are forested by *Picea engelmanni* and *Abies lasiocarpa*. The Foothills form the eastern border of the Rocky Mountains, with moderately rugged topography and lower elevation than the Rockies. Forests are most commonly *Pinus contorta* with *P. glauca*. Forest harvesting and energy development are common.

### Study design

Baited survey sites were deployed in December and sampled monthly until March, a period during which food is scarce and bait attracts mammals. Differences in logistical requirements in the Rockies versus Foothills necessitated different sampling designs. In the Rockies, we used a systematic sampling design constrained by helicopter access and avalanche risk. Sixty-six sites were placed an average of 5,727 m apart (SD = 1,574 m); 30 were sampled in 2006–2007 and the remainder in 2007–2008. In the Foothills, we deployed sites in 2005–2006 along a ca. 415-km transect, using a constrained systematic design that followed access and omitted wetlands, areas adjacent (<1 km) to main highways, and current industrial activity. Fifty-four sites were deployed, an average of 4,335 m apart (SD = 5,218 m), ca. 50 m from access roads.

### Sampling species occurrence

We used a combination of noninvasive genetic tagging (NGT) via hair sampling and camera trapping to survey mammal occurrence. NGT samples were collected using Gaucho® barbed wire (Bekaert, Brussels, Belgium) wrapped around a tree rebaited monthly with a whole beaver (ca. 10 kg). Species were identified from hair DNA (Wildlife Genetics International, Nelson, British Columbia, Canada). DNA was extracted from hairs using QIAGEN®'s DNEasy™ Tissue Kits (QIAGEN, Hilden, Germany) and analyzed to identify species using sequence-based analysis of the 16S rRNA gene of mitochondrial DNA (mtDNA; sensu Johnson and O'Brien 1997) that was then compared against a DNA reference library of all known mammal species in the region. We summed presences across 3 months to yield a 0–3 count of species occurrences at each site. NGT captured only mustelids; so in the Rockies, we also used infra-red-triggered digital camera traps (O'Connell et al. 2010). We deployed Reconyx RM30 (2006–20087) or PM30 (2007–2008) infrared-triggered digital cameras (Reconyx, Holmen, WI, USA) at all 66 sampling sites. Camera traps were effective at detecting more mammal species that visited a site. We summed each species’ presence across 3 months to yield a 0–3 count of species occurrences at each site.

### Habitat analysis

We used a LandSat thematic-mapped GIS land cover dataset incorporating a digital elevation model classified using a habitat-identification algorithm (McDermid et al. 2009). This dataset yielded 16 potential habitat types. We retained seven that were hypothesized to be of ecological importance to the study species, and which occurred sufficiently often in the study landscape to allow modeling: closed conifer forest, moderate conifer forest, open conifer forest, mixedwood forest, open wetland, upland shrubs, and upland herbaceous habitats (see McDermid et al. 2009 for descriptions). The remaining nine variables occurred very infrequently in these landscapes and were not expected to be important predictors of occurrence. We used ArcGis 9.3 (Environmental Systems Research Institute Inc., Redlands, CA, USA) Spatial Analyst to geo-reference each sampling site on the habitat GIS layer. Using spatial analysis routines (written based on Arc-View v3.× Spatial Analyst) and the Regional Analysis function of *Patch Analyst*, an extension to ArcView (http://flash.lakeheadu.ca/wrrempel/patch), we calculated the percentage of each land-cover habitat type at 20 spatial scales, consisting of a circle with radii of 250–5,000 m, in 250-m increments. Thus, holding grain constant, we analyzed 20 different extents, which we refer to as spatial scales.

### Statistical analysis

We modeled species occurrence against available habitat surrounding each sampling point using generalized linear models (Poisson errors, log link) in *R* ver. 2.10.1 (R Development Core Team, 2010). Mustelids were analyzed using the combined Foothills and Rockies dataset; other species were analyzed using only the latter. Each candidate set of models consisted of combinations of the seven habitat variables measured at a single spatial scale. For each candidate set of models – one set for each scale – we used the *step-AIC* function (MASS package for R software) to identify the best-fit model, defined as the model with the lowest (Akaike information criterion) AIC score (Burnham and Anderson 2002). AIC weights were calculated for each best-fit model, and plotted against the spatial scale at which that model was derived.

Recognizing that each spatial scale we chose may only approximate the interval in which the possibly “true” characteristic scale lies, we defined a *characteristic scale* as a single scale, or range of spatial scales, at which the species-habitat model is supported by >50% of the weight of evidence as defined by summed model AIC weights. We repeated this method for each species to determine whether a characteristic scale of selection could be identified. Where a characteristic scale was identified, we tested whether this scale was significantly explained by body mass and home-range size values (from Holling 1992) using multiple linear regression models.

To quantify patterns in spatial structure of our landscapes across scales, we calculated the variance of each habitat metric at each spatial scale and plotted these across scales (sensu Wheatley 2010).

## Results

We obtained repeat detections of *Glaucomys sabrinus* (flying squirrel), *Tamisciurus hudsonicus* (red squirrel), *Lepus americanus* (snowshoe hare), *Mustela erminea* (ermine), *M. americana* (marten), *M. pennanti* (fisher), *Gulo gulo* (wolverine), *Vulpes vulpes* (red fox), *Canis latrans* (coyote), *C. lupus* (wolf), *Lynx canadensis* (lynx), and *Puma concolor* (cougar). Mustelids were detected in both the Foothills and the Willmore, whereas all other species were only detected in the Willmore, where we had deployed infrared-triggered cameras. Detection frequency (*n*) differed among species, and consequently so did model fit (Table 1).

**Table 1:** Generalized linear models (Poisson errors) of mammal occurrence against percent cover of habitat types in the surrounding landscape. Habitat was measured at 20 different spatial scales; the best-supported model (highest AIC weight) among 20 models is shown for each species

Species common name	n*	Mass (g)**	Characteristic scale	Null deviance	df	Residual deviance	df
Cougar	8	66,508	250	43.29	65	24.72	62
Wolf	8	43,205	3,000	38.64	65	18.01	61
Flying squirrel	9	105	4,500	50.28	65	38.10	63
Coyote	11	14,061	4,250	55.54	65	34.61	63
Lynx	15	10,149	1,250	66.82	65	47.94	62
Ermine	17	81	5,000	97.47	65	63.54	63
Hare	19	1,497	5,000	72.54	65	58.78	62
Fox	21	5,193	250	77.72	65	63.56	62
Red squirrel	39	191	2,000	77.80	65	58.31	63
Fisher	48	3,118	500	188.92	119	147.88	115
Wolverine	48	12,303	5,000	183.58	119	104.94	116
Marten	62	839	4,500	164.75	119	118.26	115

* *n* = number of sites at which the species was detected.

** Masses are rounded from Holling (1992).

### Peaks in characteristic scales

Cougars, wolves, coyotes, and flying squirrels were observed at fewer than 20% of the sampling sites. Analysis of model AIC weights across scales for these species did not reveal peaks for any species but cougar. Among the eight species detected at >20% of the sites, we observed peaks in selection scale for six.

**Figure 1:**
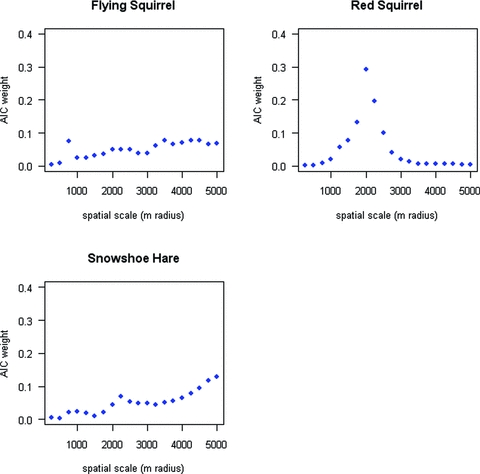
Support for models (AIC weights) of sciurid and lagomorph occurrence against habitat measured across 20 spatial scales (circles of 250– 5,000 m radii) around each sampling point.

**Figure 2:**
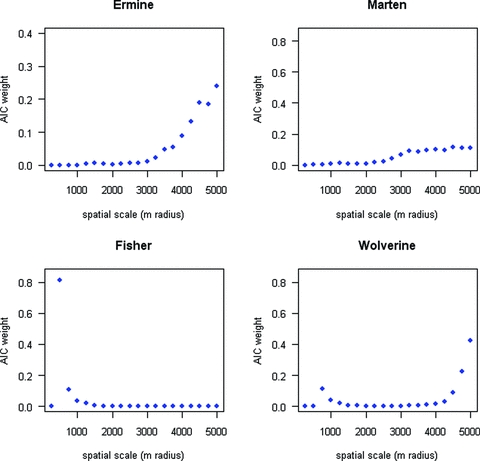
Support for models (AIC weights) of mustelid occurrence against habitat measured across 20 spatial scales (circles of 250–5,000 m radii) around each sampling point.

Among sciurids, red squirrels showed a definitive peak between 1,750 m and 2,250 m radii (Fig. 1), supported by 62.3% of the weight of evidence. Flying squirrels showed no pattern. The only lagomorph—snowshoe hares—showed a very weak multimodal pattern. Among mustelids, wolverine showed the clearest peak (Fig. 2); 64.6% of the weight of evidence suggests habitat measured at the 4,750–5,000 m scales best explains occurrence. Fisher also showed a clear pattern, as 81.4% of the weight of evidence suggests 500-m radius is the best selection scale. For ermines, 61.5% of the weight of evidence supported a characteristic scale of 4,500–5,000 m. Marten showed a much weaker pattern, as AIC weights did not exceed 0.11. Weights increased from the 2,500-m scale to the 5,000-m scale, but no distinct peak was evident.

**Figure 3:**
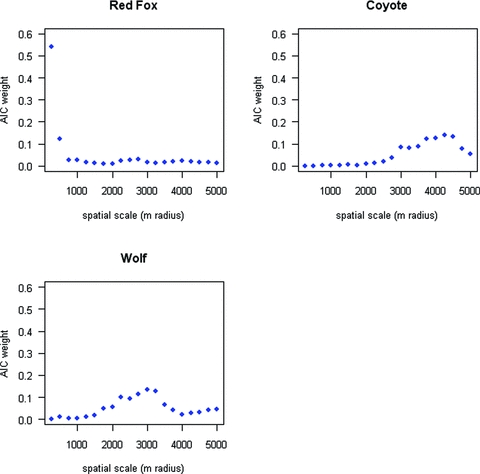
Support for models (AIC weights) of canid occurrence against habitat measured across 20 spatial scales (circles of 250–5,000 m radii) around each sampling point.

**Figure 4:**
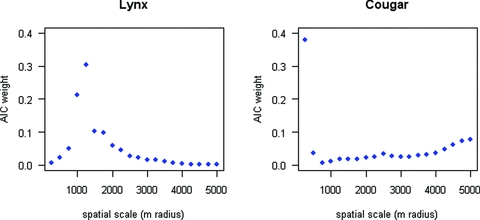
Support for models (AIC weights) of felid occurrence against habitat measured across 20 spatial scales (circles of 250–5,000 m radii) around each sampling point.

Among canids, red fox habitat selection showed a distinct, high peak at the 250-m scale (Fig. 3), supported by 66.3% of the weight of evidence. Coyote habitat selection showed a less distinct pattern. AIC weights increased from 2,750-m to 4,250-m scales, then decreased, though AIC weights did not exceed 0.14. Wolves showed a similar weak pattern, except that AIC weights increased from 1,750 to 3,000 m, and then decreased. Both felids showed distinct peaks in habitat selection scale (Fig. 4). Over half the weight of evidence (51.6%) suggested lynx selected habitat at scales between 1,000 m and 1,250 m. Habitat models for cougars peaked at 250 m (AIC weight = 0.378) and no other models were supported, but low detection and poor model fit (Table 1) suggests this model is spurious.

### Variance in habitat metrics

**Figure 5:**
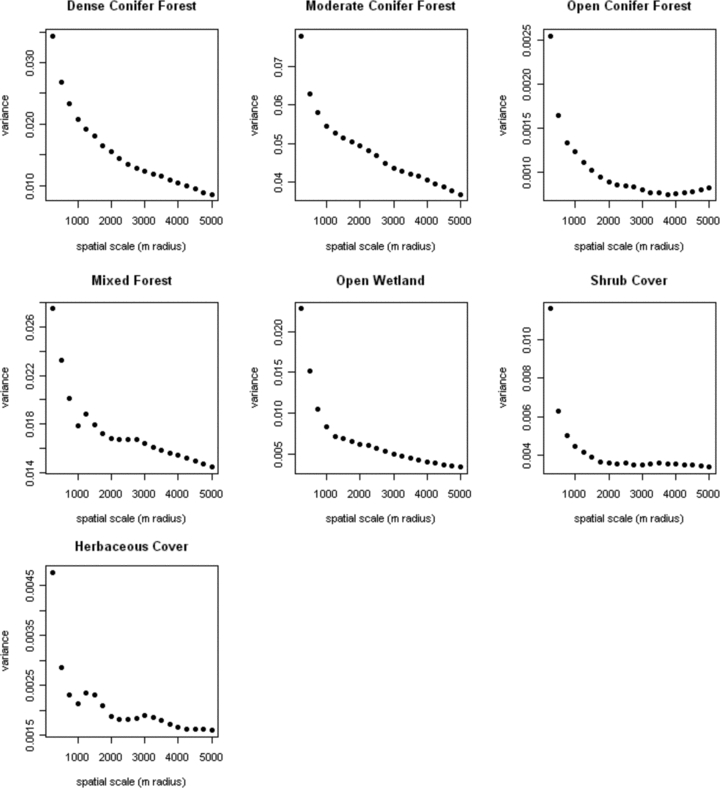
Variance in habitat metrics (percent cover of patch type) across spatial scales. Variance is highest at small scales as they tend to truncate average patch size, then decreases as scale increases.

Variance in percent cover of each habitat variable decreased with spatial scale (Fig. 5). Variance was highest at the smallest scale; in general, the variance decreased rapidly to the 1,000-m scale, and then reached an asymptote. This pattern was similar for all seven habitat variables used in the analysis. Maxima or minima in variance did not match characteristic habitat selection scales across species.

### Relationship between body mass and habitat selection scale

**Figure 6:**
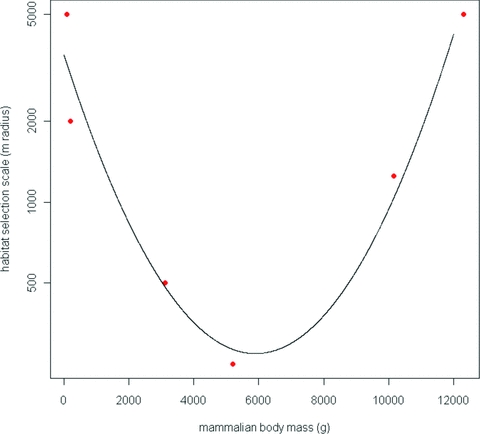
Characteristic scale of habitat selection (determined by AIC weight, see Figs. 1 and 2), log-transformed and modeled against body mass of six mammal species for which a characteristic scale was detectable. Habitat quantified at large scales best predicts both small and large mammal occurrence, whereas habitat quantified at small scales best predicts occurrence of intermediate-sized mammals.

For the six species with a defined characteristic scale of selection—red squirrel, ermine, wolverine, fisher, red fox, and lynx—home-range size (from Holling 1992) was not a significant predictor of characteristic selection scale (linear model; *P* = 0.216; *F* = 2.16; df = 1 and 4; adjusted *R*^2^ = 0.35). Contrary to our hypothesis, body mass was also not a significant predictor in the linear model (*P* = 0.853; *F* = 0.04; df = 1 and 4; adjusted *R*^2^ = 0.0097). However, the pattern of the plotted data led us to test whether the relationship between body mass and selection scale may be logarithmic and quadratic, so we modeled log(selection scale) against mass and mass-squared. This analysis suggested that a quadratic function of body mass is a significant predictor of selection scale. Selection scale decreased with increasing body mass, and then increased again (Fig. 6). Model terms are statistically significant (mass *P* = 0.0065; mass^2^*P* = 0.0056; model *P* = 0.0128; *F* = 25.97; df = 2 and 3). The model explains a high percentage of variation in the data (multiple *R*^2^ = 0.95).

## Discussion

This first explicit test for characteristic scales of selection across species demonstrates a link between body size and spatial scales of habitat selection. Several key points emerge. First, a characteristic habitat selection scale was definable for half of the 12 mammal species we studied. The marked peaks in model support at distinct scale domains for these species strongly suggest the existence of a characteristic scale of habitat selection, similar to Holland et al. (2004), Nams et al. (2006), and Mowat (2006).

The existence of a characteristic spatial scale of selection has some key theoretical implications. Our results support Wiens’ (1989) and Levin's (1992) hypothesis that ecological processes operate at characteristic scale domains. Though widely accepted, there have been few empirical tests of this hypothesis across a broad range of spatial scales (but see Holland et al. 2004; Nams et al. 2006). Our results also support their contention that not all scales serve equally well as a basis for examining ecological processes; some scales are better than others, since ecological processes manifest at some scales but not others. For example, models relating red squirrel (a conifer specialist) occurrence to open conifer forest at the 250-m scale yielded a nonsignificant relationship (*P* = 0.0963, null deviance = 77.8, residual deviance 74.6, df = 65). This same model at the 2000-m scale yielded a significant relationship (*P* = 0.000685, residual deviance = 62.3). If we test for habitat selection at the wrong scale, we fail to detect existing relationships that become observable at appropriate scales; this remains an ongoing problem, despite greater acceptance of scale dependency in habitat selection (reviewed in Wheatley and Johnson 2009). This is arguably true of all studies of scale-dependent ecological processes: study scales must be empirically modeled and matched to process scales, or risk flawed inferences and conclusions.

We found that characteristic habitat selection scales vary among species, suggesting that some scale-dependent ecological mechanism is driving habitat selection differently for each species. The importance of landscape structure for species has also been indicated by Roland and Taylor (1997), Nams et al. (2006), Mowat (2006), Schaefer and Mayor (2007), Mayor et al. (2009b), and Knegt et al. (2010a), but the mechanism is still debated. If we had observed that the same habitat variables were significant for small and large mammal species, we might suspect that a shared response to landscape pattern was driving the observed curvilinear relationship. This was not the case; different combinations of variables emerged as significant for each species. Alternatively, the variance in habitat metrics may be expected to predict the characteristic scale of habitat selection (e.g., Mayor et al. 2007, 2009b; Schaefer and Mayor 2007; Wheatley 2010; Knegt et al. 2010a). However, variance of the habitat metrics decreased from the 250-m scale to the 5,000-m scale. Scales with maximum or minimum variance are not the scales at which habitat best predicted species occurrence. Though further investigation with different landscape metrics may add insight, our results do not support the hypothesis that scale-dependent changes in habitat and landscape structure match species’ characteristic selection scales.

Instead, our analysis suggests that characteristic scales of habitat selection are related to body size. Body size has been previously implicated as a driver of spatial scales of herbivore foraging (Wilmshurst et al. 2000; Laca et al. 2010) due to constraints on rates of food intake imposed by body size (Peters 1983), and has a well-known relationship with home-range size (Haskell et al. 2002). However, all but three of our study species are carnivores, which do not forage directly on the vegetation within the habitat types we quantified. This suggests the scale-dependent response to habitat extends beyond individuals’ foraging patterns within and between food patches. Rather, species are likely responding to several concurrent ecological processes structured by heterogeneous landscapes (*sensu* Wiens et al. 1993). We suggest that the spatial scale of habitat selection is the result of a species’ interaction with the structure of its landscape, mediated by its body size.

Our conclusions are consistent with Holling's (1992) textural-discontinuity hypothesis. Holling (1992) suggested that body-size gaps represent scale domains wherein resource-patch size is too small to provide enough resources to support a body mass *x*, but patch dispersion is too great for a stride length *f(x)* to efficiently use multiple patches. Holling also suggested that within a scale domain, a species either had to be small enough to extract energy from resources attainable within the limits of its vagility, or large enough to use multiple patches. Species evolve body masses aggregating about a mean that is adapted to the characteristic resource patchiness within each scale domain (Holling 1992). Thus, the probability of a species’ occurrence in space is explained by a spatial extent related to its body size.

If body-size distributions arose from evolutionary constraints imposed by discontinuities in spatial landscape patterns, then body size may predict species’ scale-dependent response to landscape structure. We expected such a relationship between spatial scale of selection and body size to be linear, reflecting (for example) the ecological processes of landscape complementation and supplementation (Dunning et al. 1992). The quadratic relationship between body mass and selection scale that we observed was unexpected. We hypothesize that the quadratic relationship reflects individual-level responses to landscape structure for large mammals, and population-level responses for small mammals. By way of analogy, in large mammals we have detected a behavioral response, whereas in small mammals we have detected a numerical response. Within the range of spatial extents that we measured, mid-size and large mammals select denning and shelter sites, foraging patches, and home ranges based on resource availability (sensu Johnson 1980). The spatial scale at which this selection occurs is mediated by species’ body size. So, mid-size mammals select habitat that reflects resource requirements at smaller scales than do larger mammals. In contrast, for small mammals we hypothesize that landscape heterogeneity affects population processes such as dispersal, immigration, and emigration, rather than foraging (Pulliam 1988). Patch size, proximity, and heterogeneity drive population dynamics and determine probabilities of local population persistence at large scales. Individual-level resource selection likely occurs at a smaller grain (higher resolutions) than we measured.

The dichotomy in the ecological processes creating the observed patterns in scale-dependent habitat selection might result in the quadratic relationship we measured. Large scales predict small mammal occurrence through population processes; and the scale of habitat selection, through individual-level resource selection, competition, and predation, increases with increasing body size from mid-size through larger mammals. Some support for this hypothesis may already exist. Recently Brown (2007) found that body size mediated birds’ response to forest habitat patch size and isolation. Very small and very large birds responded similarly to landscape structure, whereas large and mid-sized birds responded differently. Brown (2007) attributed this pattern to different dispersal and space-use abilities among size classes, analogous to the limitations imposed by body size and stride length in nonvolant mammals.

We did not find a characteristic scale of selection for some species, for which we suggest the following potential explanations. (1) There were insufficient observations to detect a characteristic scale. This is most likely to apply to flying squirrels, coyote, wolf, and cougar. (2) While we have used a range of spatial scales to look at these relationships, we recognize that these scales could be too large for some species (e.g., flying squirrel) and not large enough for others (e.g., coyote, wolf, and cougar). (3) Some animals have multiple scales of selection. We contend that failing to detect an existing characteristic scale for species with low observation number (Type 2 error) is more likely than falsely detecting a nonexistent scale for six species (Type 1 error). The lack of a peak for marten and hares, however, requires further investigation. It is possible that a species might be selecting for different habitat variables at different scales. In fact, we would expect this to be the case (sensu Johnson 1980), as habitats are distributed differently within the study landscape, and their value likely varies with their availability and distribution. We could not include all variables at all different scales in a single model; with *n* = 120 (wolverines, fisher, marten) or *n* = 66 (all other species), the number of potential variables exceeds *n*, which Burnham and Anderson (2002) strongly recommend against. Instead, we modeled variables at a subset of scales (250; 500; 1,000; 2,000; and 4,000 m) to examine whether different variables were significant at different scales (Appendix 1). Most models failed to achieve convergence, but those that did (marten, fisher, wolverine, red squirrel) produced different significant variables at different scales as anticipated, and did not necessarily predict species’ occurrence better than single-scale models (Appendix 1). We might expect scale-dependent selection among different variables to reduce our power to find a pattern across scales, but this was not the case. The fact we did find a characteristic scale of selection for some species, despite selection of different habitat variables among scales, shows the relationship is robust.

Our results illustrate that ecological studies examining the many environmental variables driving habitat selection must be extremely cautious about spatial scale and the area that they define as *habitat*. Our results also suggest that habitat selection may have evolved in conjunction with body size; likewise, body size may have evolved to match the distribution of resources on the landscape (*sensu* Holling 1992). This relationship provides ecologists a basis for identifying the range of scales at which to conduct studies of habitat selection and a conceptual foundation for examination of the mechanisms driving species’ distribution patterns.

## Appendix

**Table:** Comparison of single-scale and multi-scale habitat selection models for mammal species in mountain regions of Alberta, Canada. The single-scale model is that with the lowest AIC score from a candidate set of single-scale models, each with habitat quantified at a spatial scale ranging from 250-m to 5000-m radii, at 250-m intervals. The multi-scale model is that with the lowest AIC score from a stepwise selection of a global model containing all habitat variables measured at 5 scales – 250 m, 500 m, 1000 m, 2000 m, and 5000 m

	Single-scale models			Multi-scale models		
		
Species	Habitat Variables+ (Scale)	Null/Residual Deviance*	AIC Score	Habitat Variables+	Null/Residual Deviance*	AIC Score
wolverine	(5000-m)	183.6 (119)	281.86	HERB_250	183.6 (119)	286.47
	DCON	105.0 (116)		SHRUB_500	95.6 (109)	
	HERB			HERB_500		
	MFOR			MCON_500		
				OWET_500		
				DCON_500		
				MCON_1000		
				MCON_2000		
				MCON_4000		
				MFOR_4000		
fisher	(500-m)	188.9 (119)	277.73	MCON_250	188.9 (119)	268.00
	DCON	147.9 (115)		DCON_250	120.1 (106)	
	SHRUB			SHRUB_500		
	MCON			OWET_500		
	OWET			DCON_500		
				SHRUB_1000		
				MCON_1000		
				MFOR_2000		
				MCON_2000		
				MCON_4000		
				OCON_4000		
				DCON_4000		
				MFOR_4000		
marten	(4500-m)	164.7 (119)	357.53	SHRUB_250	164.7 (119)	361.36
	OCON	118.3 (115)		OWET_250	108.1 (108)	
	HERB			MCON_500		
	OWET			SHRUB_500		
	MFOR			DCON_1000		
				MCON_1000		
				OCON_2000		
				MCON_2000		
				SHRUB_2000		
				MFOR_2000		
				HERB_4000		
red squirrel	(2000-m)	77.8 (65)	155.01	DCON_2000	77.8 (65)	152.52
	DCON	58.3 (63)		OCON_2000	53.8 (62)	
	OCON			DCON_4000		

* (degrees of freedom)

+ DCON = dense conifer forest; MCON = moderate conifer forest; OCON = open conifer forest; MFOR = mixed deciduous/conifer forest; OWET = open wetland; SHRIB = shrub-dominated cover; HERB = herbaceous-dominated cover. Descriptions in McDermid *et al.* (2009).
